# Zebrafish Mutants *calamity* and *catastrophe* Define Critical Pathways of Gene–Nutrient Interactions in Developmental Copper Metabolism

**DOI:** 10.1371/journal.pgen.1000261

**Published:** 2008-11-14

**Authors:** Erik C. Madsen, Jonathan D. Gitlin

**Affiliations:** Edward Mallinckrodt Department of Pediatrics, Washington University School of Medicine, St. Louis, Missouri, United States of America; University of Pennsylvania School of Medicine, United States of America

## Abstract

Nutrient availability is an important environmental variable during development that has significant effects on the metabolism, health, and viability of an organism. To understand these interactions for the nutrient copper, we used a chemical genetic screen for zebrafish mutants sensitive to developmental copper deficiency. In this screen, we isolated two mutants that define subtleties of copper metabolism. The first contains a viable hypomorphic allele of *atp7a* and results in a loss of pigmentation when exposed to mild nutritional copper deficiency. This mutant displays incompletely penetrant skeletal defects affected by developmental copper availability. The second carries an inactivating mutation in the vacuolar ATPase that causes punctate melanocytes and embryonic lethality. This mutant, *catastrophe*, is sensitive to copper deprivation revealing overlap between ion metabolic pathways. Together, the two mutants illustrate the utility of chemical genetic screens in zebrafish to elucidate the interaction of nutrient availability and genetic polymorphisms in cellular metabolism.

## Author Summary

Copper is an essential nutrient required for multiple biologic functions. Proper uptake, transport, and excretion of copper are critical for use of this metal while reducing its inherent toxicity. While several key proteins involved in this process have been identified, there are still gaps in our understanding of copper metabolism—particularly during early development. We have used zebrafish, a genetically useful animal model system, to study genetic interactions with copper deficiency during development. We treated mutant embryonic zebrafish with a chelator that reduces the level of available copper and screened for mutants that displayed a copper deficient phenotype only in the presence of the chelator. We identified and characterized two mutants that advance our understanding of copper metabolism during the early periods of development, as well as show an interaction between copper metabolism and another fundamental pathway—that of proton transport. Our results expand our knowledge of copper metabolism and illustrate the power of this type of genetic screen in zebrafish to elucidate mechanisms of nutrient metabolism.

## Introduction

Proper maternal nutrition is critical for early embryonic development. The Dutch Famine Study examined the consequences of nutrient deprivation on developmental outcome during severe food shortages near the end of the Second World War and clearly demonstrated that inadequate nutrient availability during human gestation increases the likelihood of developmental anomalies [1]. From these initial observations arose the well-recognized link between maternal folate supplementation and the suppression of neural tube defects [2]. Despite overwhelming epidemiologic data indicating the benefits of folate and other nutrient supplementation we do not fully understand the genetics of predisposition to these abnormal developmental phenotypes when faced with suboptimal nutrient levels. There are several large difficulties in the study of these processes in mammals that have prevented faster progress. The first is that the genetics of mammals has been cumbersome. The second, and more important, is that development of placental animals occurs *in utero* making rapid detection of developmental phenotypes difficult. Finally, controlling the level of nutrient available to the developing embryo cannot be done with precision as it depends both on the genetics of the mother and the embryo as well as maternal nutrition.

Copper is an essential nutrient which when absent results in severe developmental abnormalities. This is most clearly illustrated by Menkes disease (OMIM #309400), a rare X-linked disorder of copper metabolism. Patients with Menkes disease have an array of symptoms including seizures, neurodegeneration, hypopigmentation, and lax skin which result from decreased copper incorporation into critical enzymes such as dopamine-β-hydroxylase and lysyl oxidase [3],[4]. This usually fatal disease is caused by mutations in a copper transporter, *ATP7A* (NM_000052), which resides in the secretory pathway and is responsible for transport of copper into this compartment. The Menkes gene product is also responsible for placental copper transport. While patients complete *in utero* development apparently normally, it is clear from biochemical studies at birth that there are significant defects that arise from gestational copper deficiency [5].

In order to study the effects of developmental copper deprivation our lab has previously created a zebrafish model of severe copper deficiency [6]. High doses of the cell permeable copper chelator neocuproine cause embryonic zebrafish to exhibit a Menkes-like phenotype with neurodegeneration, hypopigmentation, and connective tissue defects. Isolation and cloning of the mutant *calamity*, which shared these same characteristics, revealed a loss-of-function mutation in the zebrafish orthologue of *ATP7A* (NM_001042720). In this current study we expand this model to study the effects of induced genetic alterations on the developmental response to *mild* copper deprivation. We describe two mutants sensitive to nutritional copper deficiency that illustrate the potential power of this approach to overcome the limitations of studying gene-nutrient interactions in vertebrate organisms and that define combinations of loss-of-function mutations of known ion homeostatic pathways that result in aberrant development.

## Results

### Copper Deficiency Screen

**Figure 1:**
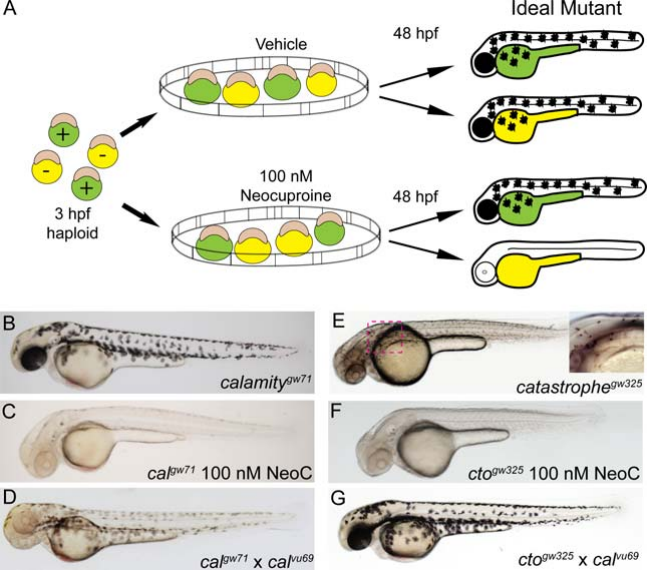
Chemical genetic screen for zebrafish mutants sensitive to copper deficiency. (A) Diagram outline of the sensitivity screen. Haploid embryos are either wild-type (green) or mutant (yellow) for any given ENU-induced mutation. These embryos were placed in vehicle or 100 nM neocuproine (neoc) at 3 hpf and allowed to develop until 48 hpf when they were screened for loss of melanin pigmentation *in drug only*. The ideal mutant is demonstrated on the right where mutant (yellow) embryos have no pigment only upon treatment with neocuproine (B–C) The first mutant isolated has full pigmentation without neocuproine (B) and loses all pigmentation upon treatment with 100 nM neocuproine (C). (D) This mutant does not complement a known allele of the mutant *calamity*, establishing it as a new allele of the same gene *atp7a*. (E–F) The second mutant has reduced, punctate pigmentation without drug treatment (E) but loses all pigmentation upon treatment with 100 nM neocuproine (F). (G) The second mutant fully complements *calamity*. Therefore we have isolated a new mutant which we have called *catastrophe*.

In order to elucidate the molecular genetics of copper metabolism we performed a forward genetic screen for zebrafish mutants with enhanced sensitivity to subthreshold copper deficiency. To control copper levels zebrafish embryos were treated with the cell permeable copper specific chelator neocuproine which has been previously shown to cause a copper-deficient phenotype including loss of pigmentation and notochord defects at a dose of 1 to 10 µM due to loss of cuproenzyme activity [6],[7]. Prior to screening, a subthreshold dose of 100 nM neocuproine was determined to cause no alteration in pigmentation in wild-type, haploid embryos. We then used this concentration of neocuproine to screen clutches of haploid embryos derived from F1 carriers of ENU-induced mutations. One half of each clutch was placed in 100 nM neocuproine at 3 hours post fertilization (hpf) and allowed to develop until 48 hpf when clutches were screened for loss of melanin pigmentation in 50% of the embryos (Figure 1A). Only those clutches which had loss of pigmentation at 100 nM neocuproine but contained at least some pigmentation when untreated were scored as mutant. In this pilot screen we examined 700 F1 females and found five potential mutants. Seven hundred mutagenized haploid genomes at an estimated single locus mutation rate of 1.1×10^−3^ represents approximately a 65–70% coverage of the genome [8]. Of the five potential mutants, four were confirmed as true mutants as defined by the transmission of the neocuproine sensitive phenotype to the offspring. One of these mutants fit the “ideal” criteria (no defect in vehicle and complete loss of pigment in 100 nM neocuproine in 50% of the haploid clutch) as illustrated in Figure 1A and subsequent analysis revealed important insight into the intersection of genetics with sub-optimal copper nutrition in early development. A second mutant reveals a role for proton transport in copper metabolism. The final two mutants were similar in phenotype to the first but full analysis has not been completed.

The first mutant isolated from the screen displayed normal melanin pigmentation when untreated but completely lost all melanin upon treatment with 100 nM neocuproine (Figure 1B, C). Crossing this mutant with *calamity^vu69^* (*cal*) which bears an inactivating mutation in the copper transport protein *atp7a* resulted in partial non-complementation. The compound heterozygote had no melanin in the developing retinal pigment epithelium (RPE) and normally distributed mild hypopigmentation over the rest of the body (Figure 1D). Based on the partial non-complementation we tentatively assigned this mutant as an allele of *calamity*, designated *gw71*.

The second mutant has a phenotype that is independent of neocuproine. Named *catastrophe*, this mutant has normally distributed melanocytes that are small and punctate (Figure 1E). *Catastrophe* (*cto*) is homozygous lethal at about 3 days post fertilization (3 dpf). The heterozygotes have no overt phenotype. In addition, *cto* homozygotes display sensitivity to copper deficiency by losing all melanin pigmentation in 100 nM neocuproine (Figure 1F). Crossing *cto* with *cal^vu69^* results in complete complementation (Figure 1G) including the observation that the double heterozygote is not more sensitive to neocuproine than *cal^vu69^* heterozygotes (data not shown). Thus, we continued our analysis on the basis that *cto* identifies a new locus involved in copper metabolism.

### A Hypomorphic Allele of *atp7a*

**Figure 2:**
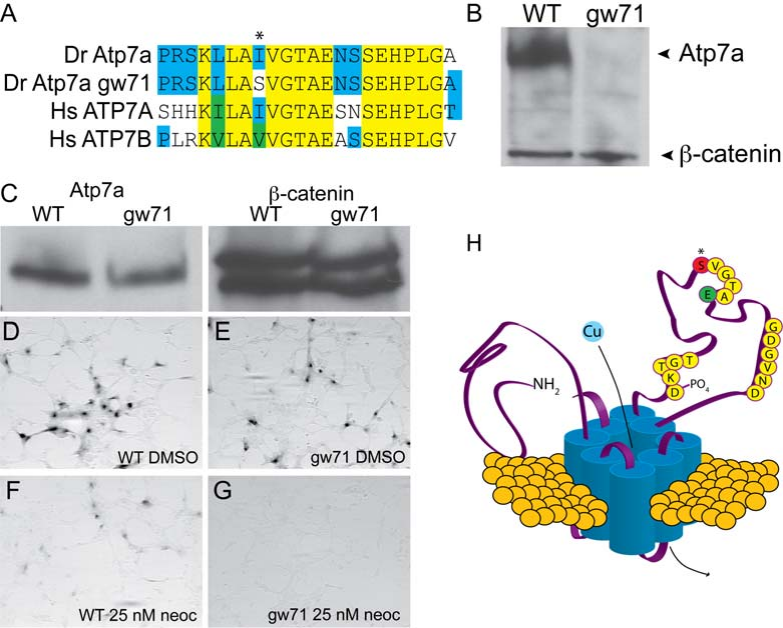
*calamity^gw71^* contains a hypomorphic allele of *atp7a*. (A) The mutation in *cal^gw71^* is a T1061S substitution in a highly conserved region of the vertebrate copper transporters and exchanges a normally hydrophobic amino acid for a hydrophilic one (asterisk). (B) This single amino acid change results in near complete loss of immunoblot-detectable protein levels. Wild-type and *gw71* mutant embryos were blotted for Atp7a using a peptide antibody to the C-terminus of the protein. β-catenin was used as a loading control. (C) Despite loss of protein in the zebrafish, transfection of an ATP7A-deficient human fibroblast cell line with either wild-type or mutant cDNA (derived from site-directed mutagensis of the wild-type) results in near equivalent expression of the zebrafish protein. β-catenin is again used as a loading control. (D–E) Both wild-type (D) and mutant (E) cDNAs are capable of producing functional protein as measured by functional tyrosinase activity in ATP7A deficient fibroblasts fixed and stained with L-DOPA. (F–G) Both wild-type (F) and mutant (G) Atp7a are sensitive to the effects of low-dose neocuproine in the above assay; however, the mutant cDNA is much more sensitive to mild copper chelation (F vs. G). (H) Model illustrating the relationship of the mutation (red, asterisk) to the known topology and functional domains of Atp7a. The mutation lies in the ATPase domain of the protein near a glutamate (green “E”) required for ATP binding and hydrolysis [10].

Chromosomal localization using the early pressure parthenogenesis method [9] placed the mutation in *cal^gw71^* (referred to below as *gw71*) near the centromere of chromosome 14, the known location of *atp7a*. Combining this data with the partial non-complementation, we hypothesized that this mutant represented a hypomorphic allele of *atp7a* and confirmed this by direct sequencing of the mRNA. Mutant *atp7a* was cloned and displayed 100% identity with the published *atp7a* sequence (NM_001042720) with the exception of a single base change present in both mutant clones, T3182G, which results in a single, non-conservative amino acid substitution, I1061S (Figure S1A). This mutation is located in a region highly conserved in copper transporting ATPases and exchanges a hydrophobic amino acid for one that is polar and hydrophilic (Figure 2A). This single amino acid change results in significant depletion of the full-length protein in mutant embryos (Figure 2B).

To verify that this was the causative mutation in *gw71*, we performed an *in vitro* activity assay for the protein using wild-type and mutant *atp7a*. Fibroblasts from patients with Menkes disease which lack functional *ATP7A* were transfected with tyrosinase in combination with either wild-type or mutant zebrafish *atp7a* created via site-directed mutagenesis of the wild-type cDNA. These fibroblasts were then treated with increasing doses of neocuproine, fixed, and stained for tyrosinase activity using L-DOPA. Activity is dependent on both *atp7a* and tyrosinase cDNAs (Figure S1B and S1C). In contrast to zebrafish mutant embryos, equal amounts of wild-type and mutant Atp7a were obtained via transfection in these fibroblasts (Figure 2D). L-DOPA staining of cells expressing mutant cDNA was only mildly reduced when compared with wild-type (Figure 2D vs. E) indicating that the mutant retains some transport function. Overnight treatment with 25 nM neocuproine resulted in complete loss of tyrosinase activity in fibroblasts transfected with mutant, but not wild-type, *atp7a* though a reduction in staining was observed with wild-type (Figure 2F, G). These data suggest that this single mutation in *atp7a* not only affects steady-state protein levels but is also capable of reducing the functional capacity of the protein, leading to sensitivity to copper deficiency.

The I1061S mutation is located in the intracellular loop which comprises the ATPase domain of the transporter (Figure 2H). Dimitriev et. al. have previously performed NMR spectroscopy on the homologous domain of the Wilson disease copper transporter, ATP7B, in the presence and absence of bound ATP and have derived from the resulting chemical shift data the residues important for ATP binding and hydrolysis [10]. We mapped the same region of Atp7a onto their model by sequence alignment (64% consensus, 49% identical) to better understand the potential effect of this mutation on protein function. The mutation in *cal^gw71^* lies five amino acids away from a critical ATP binding residue, E1064, which is highly conserved from yeast to humans (Figure 2A and Figure S1D). While a mutation of a critical residue would be expected to significantly alter ATP binding or hydrolysis, a non-conservative mutation in the region of a critical residue might be expected to only slightly alter ATP binding/hydrolysis through minor shifts in regional structure.

### *gw71* Mutants Display Post-Embryonic Sensitivity Phenotypes

**Figure 3:**
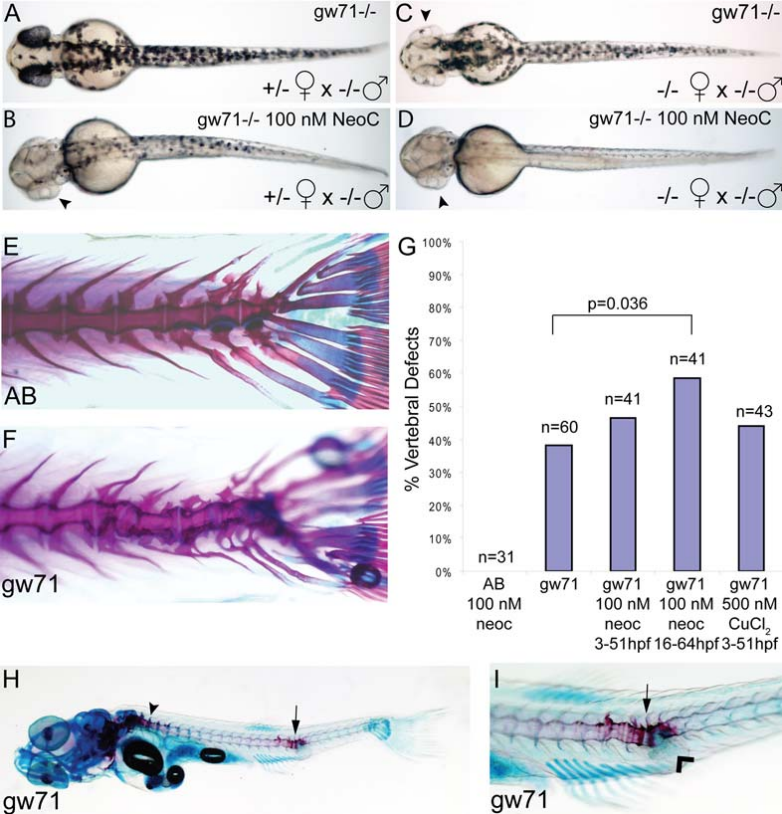
*calamity^gw71^* embryos display developmental defects that are sensitive to maternal and environmental copper availability. (A–D) Maternal effect on pigmentation in untreated *gw71* homozygous embryos. Mutant embryos derived from a heterozygous mother (A) display near normal pigmentation and have an incomplete loss of pigmentation in 100 nM neocuproine (B) most noticeable in the retina (arrowhead). Mutant embryos derived from homozygous mothers have mild hypopigmentation (C), particularly of the retina (arrowhead) and lose nearly all pigmentation in 100 nM neocuproine (D). (E–I) Partially penetrant juvenile skeletal deformities are present in *gw71* mutant fish. Wild-type (E) and *gw71* mutant (F) 21 dpf larvae were stained with alcian blue (cartilage) and alizarin red (bone) to reveal skeletal defects. In (G), embryos were untreated, treated with 100 nM neocuproine, or treated with 500 nM CuCl_2_ during the times indicated. The larvae at 21 dpf were scored according to absence or presence of a vertebral axis defect. A one-tailed Fisher exact probability test was used to calculate p-values. Only the indicated p-value was significant. (H) *gw71* mutants at an earlier stage of bone ossification display hyperossification at the location of the vertebral defect (arrow). Normal ossification is detected by alizarin red staining and begins rostrally (arrowhead). (I) A higher magification of the defect in (H) showing the hyperossification (arrow) and an outpouching of connective tissue which stains with alcian blue (arrowhead).

Because the *gw71* allele is homozygous viable, we were able to examine several post-embryonic roles for *atp7a*. Adult homozygous mutant zebrafish placed in varying doses of neocuproine did not display an overt sensitivity phenotype (data not shown). However, further study revealed a maternal effect of this mutation on embryonic copper metabolism. Homozygous mutant embryos derived from heterozygous females had a normal quantity and distribution of pigmentation that was partially sensitive to 100 nM neocuproine which abolished pigment in the retinal pigment epithelium (RPE) and reduced pigment over the body of the fish (Figure 3A, B). In contrast, homozygous mutant embryos derived from homozygous mutant females had no pigment in the RPE and reduced pigment over the body; treatment of these embryos with 100 nM neocuproine completely abolished pigmentation throughout the embryo (Figure 3C, D). The effect of the mother's genotype on the embryonic phenotype indicates that though not overt, the adult homozygous mutant does have defects in copper metabolism demonstrated by a nutrient-deficient state in the offspring. Thus the sensitivity of the embryo to neocuproine is due not only to aberrant embryonic copper metabolism, as the embryos from heterozygous mothers are sensitive to copper deficiency, but also to a deficient maternal loading of copper into the egg as the phenotype is exacerbated by maternal homozygosity.

The importance of optimal copper nutrition during development is further illustrated by the presence of vertebral skeletal defects in homozygous mutants. Homozygous mutant embryos were stained at 21 dpf with alcian blue/alizarin red to reveal bone and cartilage respectively. These were compared with wild-type syngeneic age-matched controls raised in the same manner. The wild-type fish had straight vertebral columns along the entire length with long, straight bony processes extending from each vertebra (Figure 3E). In contrast, homozygous *gw71* fish displayed variable vertebral defects, most often a significant warping of the bony structures in the caudal-most region of the column caused by irregular length of vertebrae and defects in the joint angles (Figure 3F). In addition the bony processes were also shortened and bent. Consistent with the observations in embryos that the mutation in *gw71* brings the homozygous embryo close to, but not over, a threshold for copper deficiency, the persistent skeletal defects in the juvenile fish were not fully penetrant. Whereas wild-type fish had no vertebral defects (n = 26), a significant number (38%, n = 60) of the *gw71* fish contained defects (Figure 3G).

Incomplete penetrance of the defect in the homozygous mutant fish could be attributed to either separate subtle genetic interactions or to variable nutrient availability. We hypothesized that if the penetrance of the defects were based on nutrient availability then reducing the nutrient levels would worsen the defects and increase the penetrance and vice versa. We thus took *gw71* mutant embryos and placed them in either normal egg water or egg water supplemented with 100 nM neocuproine or 500 nM CuCl_2_ from 3 to 51 hpf (48 hour exposure). In addition, two separate groups of embryos were treated with neocuproine from 16 to 64 hpf and from 30 to 78 hpf to determine if there was a window of developmental time critical for the genesis of later defects. At 21 dpf the larvae were stained with alcian blue/alizarin red and scored for the presence or absence of vertebral defects (Figure 3G). Untreated wild-type embryos (not shown) or wild-type embryos treated with 100 nM neocuproine from 3–51 hpf had no perceptible skeletal defects. Thirty-eight percent of *gw71* embryos had skeletal defects and this number was not significantly affected by treatment with 100 nM neocuproine or 500 nM CuCl_2_ from 3–51 hpf. However, there was a 50% increase in the number of skeletal defects in *gw71* embryos treated with 100 nM neocuproine from 16 to 64 hpf. The larvae treated with 100 nM neocuproine from 30 to 78 hpf died approximately 8 dpf from an unidentified cause. These results indicate an increasing sensitivity to mild copper deprivation as the embryo develops in the first 16–72 hrs. Further experimentation with smaller, more discrete treatment times might allow the determination of any developmental window required for the effects of copper on vertebral axis formation.

In addition to the presence of vertebral skeletal defects in fully ossified skeletons, larvae at earlier stages of development displayed hyperossification of vertebrae adjacent to defects in the vertebral column (Figure 3H, I). Normal zebrafish bone ossification begins rostrally and generally proceeds caudally with the exception of the caudal fin vertebrae [11]. In *gw71* this pattern is maintained (arrowhead in Figure 3H) except for areas containing defects (arrow in 3H). The defects affected the joints between vertebrae and had differing degrees of connective tissue bulges which partially stained with alcian blue indicating the presence of some cartilaginous tissue in these defects (Figure 3H arrowhead).

### *catastrophe* Contains a Defect in Proton Transport

**Figure 4:**
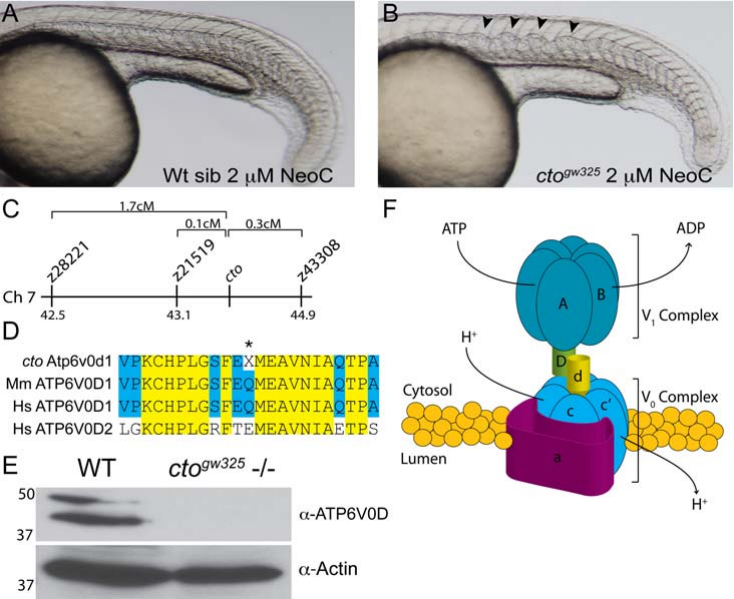
*catastrophe^gw325^* contains a copper sensitive mutation in the vacuolar (H^+^) ATPase Atp6. (A–B) *cto* embryos are globally sensitive to copper deficiency. Wild-type embryos (A) in 2 µM neocuproine do not display notochord defects. In contrast, *cto* embryos (B) placed in this same dose of neocuproine have significant distortion of the notochord in a pattern consistent with loss of lysyl oxidase activity [12]. (C) Meotic mapping placed the *cto* mutation between markers z21519 (43.1cM) and z43308 (44.9cM) on chromosome 7. (D) Atp6v0d1 is highly conserved between zebrafish and mammals and is easily differentiated from ATP6V0D2 present in humans. The amino acid Q136 is changed to a stop in the mutant (asterisk). (E) The mutation in *cto* abolishes expression of the full length protein. Immunoblot analysis using a C-terminal polyclonal antibody shows no recognition of the 40 kD band in 48 hpf *cto* embryos. The identity of the band at 50 kD is unknown. Actin was used as a loading control (lower panel). (F) Model of the proposed quaternary structure of Atp6. The lower-case d subunit (yellow) forms part of a connecting stalk between the V1 and V0 subunits the presence of which is required for proper formation of the entire transporter [19].

Before mapping the *catastrophe* mutant it was important to determine the extent of the defect in copper metabolism. The loss of pigmentation in the mutants could result from toxicity in a “two-hit” model whereby the mutation damages melanocytes and the drug acts to further affect these already sick cells. Therefore we examined the sensitivity of the mutant to another copper-dependent process–notochord formation. Notochord formation requires the action of the cuproenzyme lysyl oxidase and its family members. Both reduction in lysyl oxidase levels and copper chelation result in wavy, distorted notochords [12]. Placing *cto* mutants in 2 µM neocuproine at 3 hpf resulted in wavy notochords in the mutant embryos at 24 hpf while having no effect on heterozygous or wild-type embryos (Figure 4A, B). This experiment indicates that the mutation in *cto* causes a global defect in copper metabolism and is not limited to melanocytes.

The mutation in *cto* was localized to chromosome 7 and further mapping reduced the region of interest to an approximately 1 Mbp region between markers z21519 and z43308 (Figure 4C). It was possible to assemble a nearly complete BAC contig between these markers using database BAC sequences (www.sanger.ac.uk/Projects/D_rerio/). This contig was scanned for potential genes using the FGENESH program (www.softberry.com) and comparing to the Ensembl database (www.ensembl.org). A list of candidate genes was generated from this comparison. To further refine the list, a database of zebrafish insertional mutants was scanned for mutants displaying a similar melanocyte phenotype [13]. Approximately 6 mutants in this database had punctate melanocytes, 5 of which had insertions in genes encoding subunits of the vacuolar (H^+^) ATPase (Atp6) (NM_199620). As the critical region in *cto* contained the d subunit of the V0 complex of the vacuolar ATPase we cloned and sequenced this cDNA in the *catastrophe* mutants. A single base pair change C406T present in the mutant resulted in a premature stop codon, Q136X (Figure S2). Sequence alignment with the human sequences (NM_004691) revealed a highly conserved protein sequence (94% identical) that most closely aligned with the d1 subunit (Figure 4D). Further database searches did not reveal a second d1 subunit in zebrafish.

The significant identity between the human and zebrafish protein sequences allowed us to use an antibody directed against human ATP6V0D1 to examine the steady state levels of protein. We hypothesized that the early stop codon would result in a significant decrease in protein levels. Indeed, in 48 hpf embryos there is a near total reduction in Atp6v0d1 protein as compared with wild-type embryos (Figure 4E). Total loss of this highly conserved and essential protein (see below) may be the cause of the *catastrophe* phenotype; however, there remains some possibility that another, tightly linked mutation may contribute to the observed phenotype. Based on significant experimentation in yeast a proposed quaternary structure for the vacuolar ATPase complex has emerged (Figure 4F) [14]–[18]. In this model, the two main subcomplexes, V0 and V1 have complementary functions of proton translocation and ATP hydrolysis respectively. The complexes are connected through several stalk subunits, v1d, v0d, and v1f (not shown). Loss of these connecting subunits in yeast results in total loss of activity of the complex [19]. Thus in *catastrophe*, the loss of the v0d subunit would be predicted to result in complete loss of proton translocation throughout the embryo.

### *catastrophe* Is Sensitive to Pharmacologic Inhibition of Proton Transport

**Figure 5:**
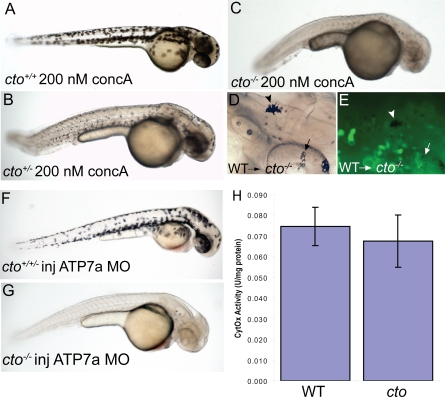
*cto* contains a concanamycin A sensitive, cell autonomous defect which affects secretory pathway copper transport. (A–C) *cto* gene dosage alters sensitivity to concanamycin A (concA) an inhibitor of Atp6. Wild-type fish have no phenotypic response when incubated in 200 nM concanamycin A beginning at 24 hpf (A). Embryos heterozygous for *cto* are sensitive to this same dose of concA, resulting in punctate melanocytes (B). ConcA exacerbates the phenotype of *catastrophe* homozygotes resulting in total loss of pigmentation and increased degenerative appearance (C). (D–E) The defect in *cto* is cell autonomous both in epidermal and retinal pigment epithelial cells. Wild-type, GFP-positive cells were transplanted into *cto* mutant embryos at the 1000 cell stage and allowed to develop to 48 hpf. Robustly pigmented melanocytes with normal size and shape can be seen sparsely distributed throughout the epidermis (D, arrowhead) and retinal pigment epithelium (arrow, D). The epidermal melanocyte does not have visible GFP but is not surrounded by GFP-positive cells (E, arrowhead). The RPE cells have a central area of GFP-positivity (E, arrow) Other areas are GFP positive without melanin pigment. (F–G) *cto* homozygotes but not heterozygotes or wild-type embryos are sensitive to *atp7a* morpholino injection. At a sensitizing dose of morpholino that does not affect wild-type/heterozygotes (F), homozygous *cto* embryos lose all pigmentation (G). (H) Cytochrome c oxidase activity is not reduced in *cto* embryos. Activity was normalized to protein levels in each sample. Three independent samples were prepared from three groups of embryos and the standard deviation of the three experiments is shown.

If the defect in *catastrophe* is loss of Atp6 function the heterozygotes might be sensitive to pharmacologic inhibition of this transporter. Consistent with this, wild-type embryos placed at 24 hpf in 200 nM concanamycin A, a potent and specific inhibitor of Atp6 [20], showed no apparent phenotype at 48 hpf (Figure 5A). However, treatment of embryos heterozygous for *cto* resulted in punctate melanocytes and CNS degeneration, resembling the mutant (Figure 5B). The mutants themselves appeared qualitatively worse, with further reductions in melanocyte pigmentation and worsening of the degenerative appearance (Figure 5C).

### Secretory Pathway Copper Transport Is Altered in *catastrophe*

While it is apparent that loss of Atp6 results in altered cuproenzyme activity for two enzymes in the secretory pathway, it is unclear which step of global copper transport is affected in *cto* embryos. To address this we performed transplant experiments to determine the cell autonomy of the defect. Wild-type cells from *actin::GFP* transgenic zebrafish were transplanted into *cto* embryos and examined at 48 hpf for pigmented cells and GFP expression. Transplantation resulted in a few well-pigmented and stellate melanocytes over the head and body as well as clusters of pigmented retinal epithelial cells (Figure 5D). These same embryos were mosaic for GFP expression (Figure 5E). In body melanocytes the melanin obscured GFP fluorescence (Figure 5E arrowhead). In contrast, the retinal pigment epithelial melanocytes display GFP fluorescence in the central area not covered by melanin (Figure 5E arrow). From this we make two observations: First, the melanized melanocytes are derived from wild-type donor cells, and secondly, that nearby wild-type epidermal cells are not required for normal melanin pigmentation nor stellate appearance (Figure 5E arrowhead). Thus copper metabolism must not be significantly disrupted on an organismal level, as these wild-type melanocytes in a mutant host still receive adequate copper for normal pigmentation. Also, the stellate appearance indicates that the defect that causes punctate pigment cells in *cto* is also cell-autonomous.

The transplant experiment addresses delivery of copper to each cell, but the uptake or distribution of copper within the individual cell could also be affected in *cto* embryos. We hypothesized that disruption of the transporter responsible for secretory pathway acidification would result in defects in copper metabolism in this compartment. To test this we examined the sensitivity of *cto* embryos to partial loss of Atp7a through the use of a morpholino. Previous work from our laboratory has demonstrated that melanin synthesis following loss of Atp7a is also cell-autonomous in the melanocyte indicating that knock-down of Atp7a will allow interrogation of the pathway on a cellular rather than organismal level [6]. Injection of a splice morpholino previously shown to result in a copper deficient phenotype at a dose that does not cause pigmentation defects in wild-type or heterozygous embryos (Figure 5F) causes total loss of melanin pigmentation in *cto* embryos (Figure 5G). Thus *cto* embryos are sensitive to loss of the secretory pathway copper transporter, Atp7a. Embryos heterozygous for the *cto* mutation did not show sensitivity to the Atp7a morpholino indicating that near complete loss of Atp6 activity is required to sensitize to alterations in copper metabolism. At the same time, the cytochrome oxidase activity of mitochondria derived from *cto* embryos is no different from wild-type indicating that copper delivery to mitochondria is normal and that the defect in copper metabolism in *cto* embryos is limited to the secretory compartment (Figure 5H).

### Subcellular Morphology and Melanosome Formation Is Altered in *catastrophe*

**Figure 6:**
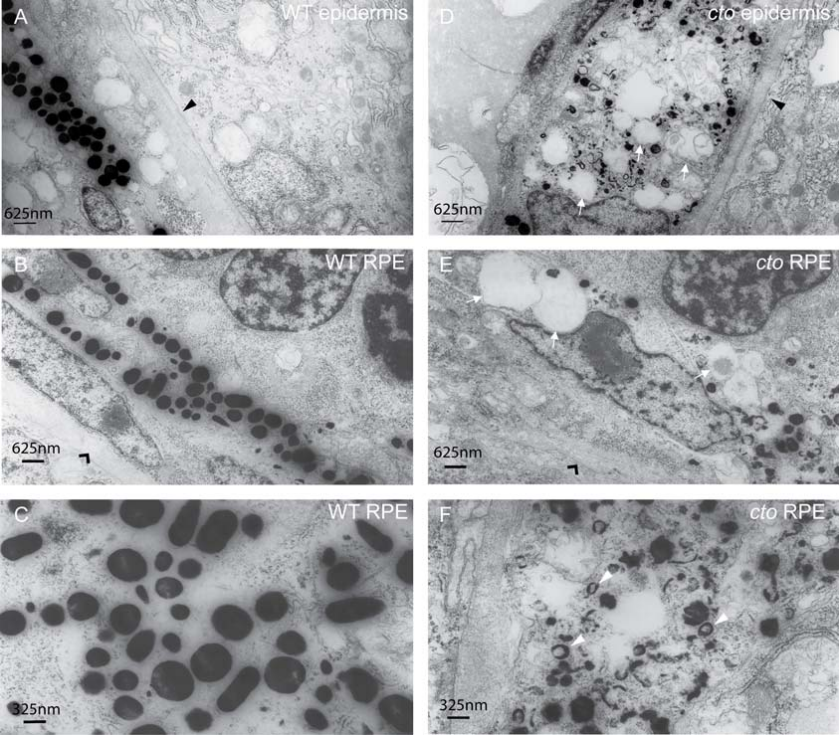
*cto* melanocytes have significant ultrastructural defects. (A–B) Wild-type epidermal (A) and retinal pigment epithelial (RPE, B) melanocytes are elongated and thin and contain many large, densely pigmented melanosomes. The epithelial basement membrane is indicated by a black triangle in (A) and the RPE basement membrane by a black arrowhead in (B). (C) A higher magnification of wild-type melanosomes showing significant pigmentation and ellipsoid shape when cut longitudinally. (D–E) *cto* mutant epidermal (D) and RPE (E) melanocytes showing rounded, poorly pigmented cells that contain numerous large, empty vesicles (white arrows) The basement membranes are indicated as in (A and B). (F) A higher magnification of RPE melanosomes showing the diverse array of immature, poorly pigmented vesicles. The white arrowheads point to multi-lamellar, melanin filled vesicles which are identical to the melanin positive multi-vesicular bodies seen in the *cappucino* mouse (see text).

The vacuolar ATPase has been implicated in diverse trafficking events within the cell and inhibition of this protein results in altered ion homeostasis, disrupted membrane trafficking, defective acid secretion, deficient protein degradation, and loss of protein sorting, endosomal recycling, and vesicular secretion [21]–[27]. To examine the effect of loss of this protein on cellular morphology, specifically melanocytes, we performed transmission electron microscopy focusing on the pigmented cells. Thin (500 nm) plastic sections of 48 hpf embryos stained with toluidine blue did not demonstrate any further gross defects in organismal or cellular morphology beyond those observed in the pigmented cells both of the epidermis and the retinal pigment epithelium (data not shown). Upon examination by electron microscopy in wild-type embryos both epidermal pigment cells as well as retinal pigment epithelial cells at 48 hpf display dark, uniformly round or ellipsoid melanosomes distributed throughout flat melanocytes (Figure 6A–C). In contrast, the melanocytes of *cto* embryos are rounded and contain few fully melanized melanosomes, many large vacuolated structures and small vesicles surrounded by rings of melanin pigment (Figure 6D–F). These latter structures have been identified as multi-vesicular bodies, the accumulation of which is reminiscent of early blocks in melanosome maturation found in the *cappuccino*, *pallid*, *ruby-eye 2*, and *reduced pigmentation* mice which are all models of Hermansky-Pudlak syndrome and have specific early defects in melanosome biogenesis [28]. Thus among other abnormalities loss of proton transport results in early blocks in melanosome maturation. It is interesting to note that there remains active tyrosinase which produces some melanin in these aberrant structures despite the loss of the proton transporting ATPase (Figure 6F).

## Discussion

### Genetic Screen for Gene-Nutrient Interactions

In this work we have used the power of forward genetic screens combined with the ease of *ex utero* nutrient level manipulation accessible with the zebrafish to study the relationship between specific genetic alterations, the levels of the essential nutrient copper, and their combined effects on the developmental phenotype of the embryo. From these experiments we have derived a nutrient-sensitive allele of a known copper transporter that results in a juvenile skeletal phenotype. We have also implicated the vacuolar proton pump in vertebrate copper metabolism and interconnect two ion transport proteins whose individual effects on the other would not otherwise have been appreciated.

The *ex utero* development of zebrafish provides an opportunity for manipulating the developmental levels of nutrients. Much success has been achieved in yeast using large libraries of compounds coupled with known deletion mutants to define the roles of many of the yeast proteins in cellular biology and metabolism [29],[30]. One major advantage of yeast is the ability to absolutely control the levels of different nutrients and pharmacologic compounds and to screen large numbers at a time; however, yeast lack the complexity necessary to extend such findings to multi-cellular organisms and ultimately to understand human biology for the treatment of disease. Our work shows that the zebrafish model system can fill the niche in extending the principles of the chemical genetic screen to a vertebrate organism. Zebrafish retain the advantage of environmental exposure control while only slightly reducing the ability to screen large numbers. They also provide a system with more complex phenotypes to be examined which can then be brought back to the study of the underlying cell biology of a multi-cellular organism, particularly as the genome sequence and rapid mapping techniques improve.

### Nutrient-Sensitive Hypomorphic Allele

The first mutant which was isolated from our screen was a hypomorphic allele of *atp7a*. Animals bearing this allele have a normal pigmentation and notochord phenotype at 48 hpf but are sensitive to mild copper deficiency thus indicating that transporter function was impaired. This mutation reduced the protein levels to below the detection limits of our immunoblot demonstrating that only a fraction of wild-type protein expression is necessary to maintain a near-normal phenotype. This is consistent with our previous observations where very minor changes in Atp7a protein levels resulted in significant rescue of the *calamity* phenotype [31]. Also, the increase in severity of the *cal^vu69^* allele upon incubation with neocuproine demonstrates that even in this model of severe Menkes disease, there is still residual protein function without detectable expression [6],[31]. Interestingly, when the *gw71* protein was overexpressed in cell culture fibroblasts it was fully capable of loading copper into the secretory pathway as evidenced by the robust tyrosinase activity; yet, at the same time there was a clear sensitivity of this mutant transporter to copper levels.

This mutant allele is not the first hypomorphic allele of *atp7a*. A less severe form of Menkes disease, Occipital Horn Syndrome, is also caused by mutations in *atp7a*. Children with this disease have many clinical problems similar to Menkes disease; however, as this syndrome is not fatal in early life other abnormalities can be appreciated including skeletal defects such as deforming hyperostosis and kyphoscoliosis [32]. In this context the *gw71* mutant provides several important advances. First, within the screen itself it provides proof-of-concept that the screen design will result in the identification of critical proteins involved in copper transport and metabolism. Second, the *gw71* allele is both viable and fertile which itself provides distinct advantages. Third, this allele demonstrates that only a fraction of wild-type levels of Atp7a protein are required for near-normal pigmentation and notochord formation, a result suggested by previous experiments [31]. Fourth, this mutant expands the hierarchy of copper metabolism previously described [6]. The differential effect on retinal pigment epithelial melanin versus the body pigmentation seen under a variety of genetic and environmental manipulations (Compare Figures 1D, 3B, and 3D) demonstrates an increased sensitivity of the RPE to derangements of copper metabolism. Fifth, the *gw71* mutant displays an incompletely penetrant developmental hyperostosis phenotype which is easily detected. The proximal etiology of these defects is unknown. It may be related to lysyl oxidase activity which is important for zebrafish notochord development and is sensitive to nutritional copper status [12]. The increase in penetrance with copper chelation suggests that the variability may be due to nutritional differences. The lack of rescue observed with copper supplementation could be due to an inability of this ion to be translocated by the mutant Atp7a protein to the proper compartment. Alternatively, lack of rescue with copper could point to residual genetic heterogeneity leading to phenotypic differences. Whichever is the case, this aspect of the mutant phenotype may provide a model to further our understanding of this poorly understood defect. The viability of this mutant would allow a modifier screen to find mutations responsible for different aspects of the copper deficient phenotype as well as to detect any genetic variability leading to the incomplete penetrance observed in the mutant.

### Intersection of Two Ion-Transporting Pathways

Our second mutant contains an inactivating mutation in the vacuolar (H^+^) ATPase subunit, Atp6v0d1. While abolition of this protein results in loss of proton transport into the secretory pathway, the embryo is capable of developing relatively normally to about 48 hpf when defects become visibly apparent. This lag is most likely due to the persistence of maternal protein and mRNA. At this time point the changes in melanin pigmentation patterns signal the visible presence of defects in proton transport. Grossly the melanocytes become punctate which, upon ultrastructure analysis, is shown to be a loss of mature melanosomes and a rounding of the cell body with vacuolization. The observed relationship between lack of melanosome formation and cellular morphology is not understood but may suggest a toxic effect of inappropriate melanization in the multi-vesicular bodies seen with electron microscopy or may be due to a particular sensitivity of melanocytes to loss of proton transport. As it has been shown that the vacuolar ATPase is important for vesicular trafficking and endocytosis [21],[22], the distinct disruption of planar morphology in *cto* melanocytes may also be due to defects in these processes.

The sensitivity to copper deficiency of the remaining melanin implicates proton transport in the homeostasis of copper metabolism. That the notochord is equally sensitive to reduced copper demonstrates that the defect is not limited to the melanocyte, but rather that there is a universal decrease in the ability of copper to adequately reach secretory cuproenzymes. Since the effect on copper metabolism in *cto* mutants is only revealed in the context of sub-threshold copper nutrition, without a screen of this nature, this inter-relationship of two ion transport pathways in the vertebrate organism would never have been appreciated.

There are two models which could explain the defect in cuproenzyme function when proton transport is compromised. The first is that an acidic pH is important for copper incorporation into the nascent cuproproteins within the secretory pathway. The second model is that a proton gradient is required for copper transport, to balance the charge transfer across the vesicular membrane. These models are not mutually exclusive and a combination of the two could result in the final phenotype.

The data presented in this paper demonstrate the power of the zebrafish model system to examine gene-nutrient interactions as well as to delineate basic cell biologic pathways. Continuing with this methodology will provide more insight into the biology of copper metabolism in a vertebrate organism. It is easy to see how screens in zebrafish similar to the one we describe have the potential to investigate the genetics of not only copper or folate metabolism, but also that this approach could be easily extended to an array of other nutrients.

## Materials and Methods

### Zebrafish Maintenance

Zebrafish were maintained in the Washington University Department of Pediatrics zebrafish facility according to institutional guidelines supervised by the Division of Comparative Medicine.

### Mutagenesis, Screen, and Mapping

The specific alterations of these well-characterized techniques are available in Text S1.

### Immunoblot

Mutant 48 hpf embryos were identified phenotypically. Twenty to thirty embryos were manually dechorionated and de-yolked, lysed in 75 µL RIPA buffer containing 10 µL/mL Protease Inhibitor Cocktail III (Calbiochem). Unlysed material was removed by centrifugation at 1000× g for 5 minutes. For Atp7a, 50–100 µg of lysate in Laemmli buffer with 10% β-mercaptoethanol, heating for 5 min at 65°C (not fully reducing conditions) was loaded on a 6% SDS-polyacrylamide gel. The protein was transferred to nitrocellulose and blotted for Atp7a using a custom polyclonal antibody raised against a C-terminal peptide [31]. For Atp6v0d1, 30–40 µg lysate in Laemmli buffer with 10% β-ME heated to 70°C for 5 minutes was loaded on a 12% SDS-polyacrylamide gel. The transferred protein was blotted for Atp6v0d1 using a mouse polyclonal raised to human recombinant protein at 1∶1000 dilution (Abnova Corp). Other antibodies: Actin (Sigma) 1∶5000, β-catenin (BD Biosciences) 1∶1000.

### Me344 Cell Culture

The Menkes patient fibroblast cell line Me344 (gift of Mick Petris) was maintained in 10% FBS/DMEM with Pen/Strep/Glut. Transfections were carried out on coverslips using Lipofectamine 2000 (Invitrogen) at a ratio of Lipo2k∶DNA of 2.5 for 3 hours in Optimem (Invitrogen). The media was then replaced with 1% FBS/DMEM/PSG. Neocuproine was added in DMSO to the indicated concentration and the cells incubated overnight.

### L-DOPA Staining

Performed as previously described [33].

### Alcian Blue/Alizarin Red Stain

Twenty-one dpf juvenile zebrafish were fixed overnight in 4% PFA in PBS and stained as previously described [34].

### Transplantation

Approximately 50–100 cells were extracted from wild-type (AB) embryos at the 1000 cell stage and placed in mutant embryos of the same age using a micromanipulator syringe and glass needle as described previously [35].

### Morpholino Injection

The *atp7a* splice morpholino e7 (TGACAACATTAACATTCATACCCTG) [31] was injected at a dose of 965 pg/embryo at the 1 cell stage in 10% phenol red. At 48 hpf the injected embryos were scored for pigmentation and genotyped.

### Cytochrome C Oxidase Activity Assay

A crude mitochondrial fraction was prepared from groups of 45 embryos at 52 hpf by homogenizing in 250 mM sucrose, 10 mM Tris pH 7.4 with a loose-fitting glass-glass tissue homogenizer. The homogenate was spun at 700× g for 10 minutes. The supernatant from this spin was centrifuged at 23,000× g for 20 min to form a pellet containing mitochondria and large vesicles. The pellet was resuspended in 150 µL of sucrose buffer with protease inhibitors and n-dodecyl-3-D-maltoside was added to 1 mM and incubated for 10 minutes at 25°C. Cytochrome c oxidase activity was monitored by measuring the decrease in absorption of ferrocytochrome c at 550 nm using the protocol described for the Cytocox assay kit (Sigma, USA).

### Transmission Electron Microscopy

Performed as described previously [12].

## Supporting Information

Figure S1 (A) Sequencing of the *atp7a* cDNA in *cal^gw71^* mutant embryos reveals a single non-synonymous nucleotide change T3182G which causes a non-conservative amino acid substitution T1061S. (B) Transfection of tyrosinase only into Me344 cells does not result in any appreciable tyrosinase activity. (C) Transfection of *atp7a* only into Me344 cells also does not result in L-DOPA oxidase activity. This activity is specific to tyrosinase expression. (D) Alignment of a small region of *atp7a* containing the mutation in *gw71* (arrowhead) and the highly conserved glutamate (asterisk) observed to be important for ATP binding/hydrolysis. This glutamate is fully conserved from fungus to humans.
(5.94 MB TIF)

Figure S2 Sequencing of the cDNA of *atp6v0d1* which lies near the *cto* locus in revealed a single nucleotide change that creates an early stop codon.
(0.43 MB TIF)

Text S1 Supplemental methods.
(0.03 MB DOC)
