# Supplementary figures and images for: Zebrafish Mutants calamity and catastrophe Define Critical Pathways of Gene–Nutrient Interactions in Developmental Copper Metabolism
Source: PLoS Genet. 2008 Nov 14;4(11):e1000261. doi: 10.1371/journal.pgen.1000261 (PMC2576455; doi:10.1371/journal.pgen.1000261)

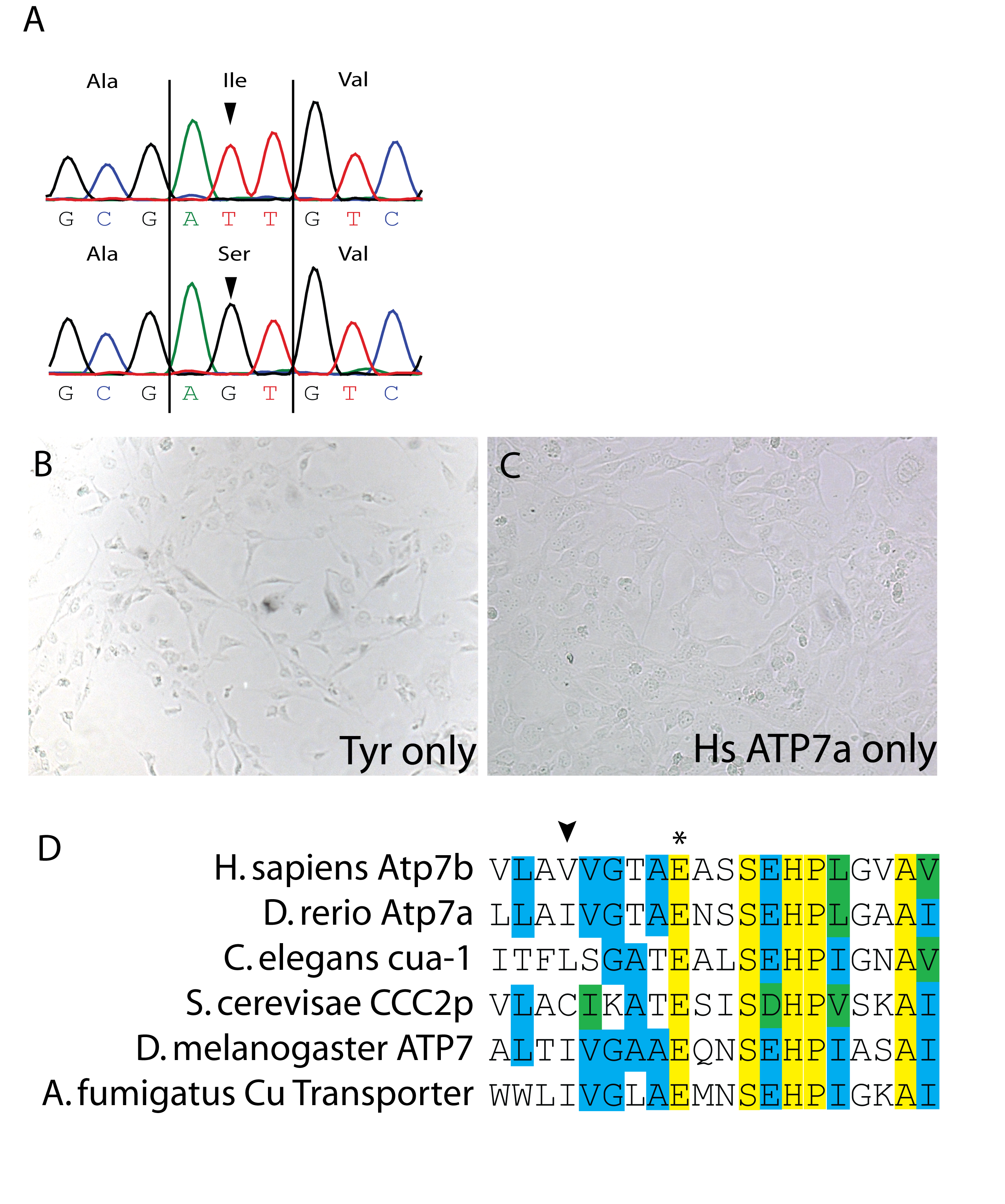

Supplement: Figure S1 — (A) Sequencing of the atp7a cDNA in calgw71 mutant embryos reveals a single non-synonymous nucleotide change T3182G which causes a non-conservative amino acid substitution T1061S. (B) Transfection of tyrosinase only into Me344 cells does not result in any appreciable tyrosinase activity. (C) Transfection of atp7a only into Me344 cells also does not result in L-DOPA oxidase activity. This activity is specific to tyrosinase expression. (D) Alignment of a small region of atp7a containing the mutation in gw71 (arrowhead) and the highly conserved glutamate (asterisk) observed to be important for ATP binding/hydrolysis. This glutamate is fully conserved from fungus to humans. (5.94 MB TIF) [file pgen.1000261.s001.tif]

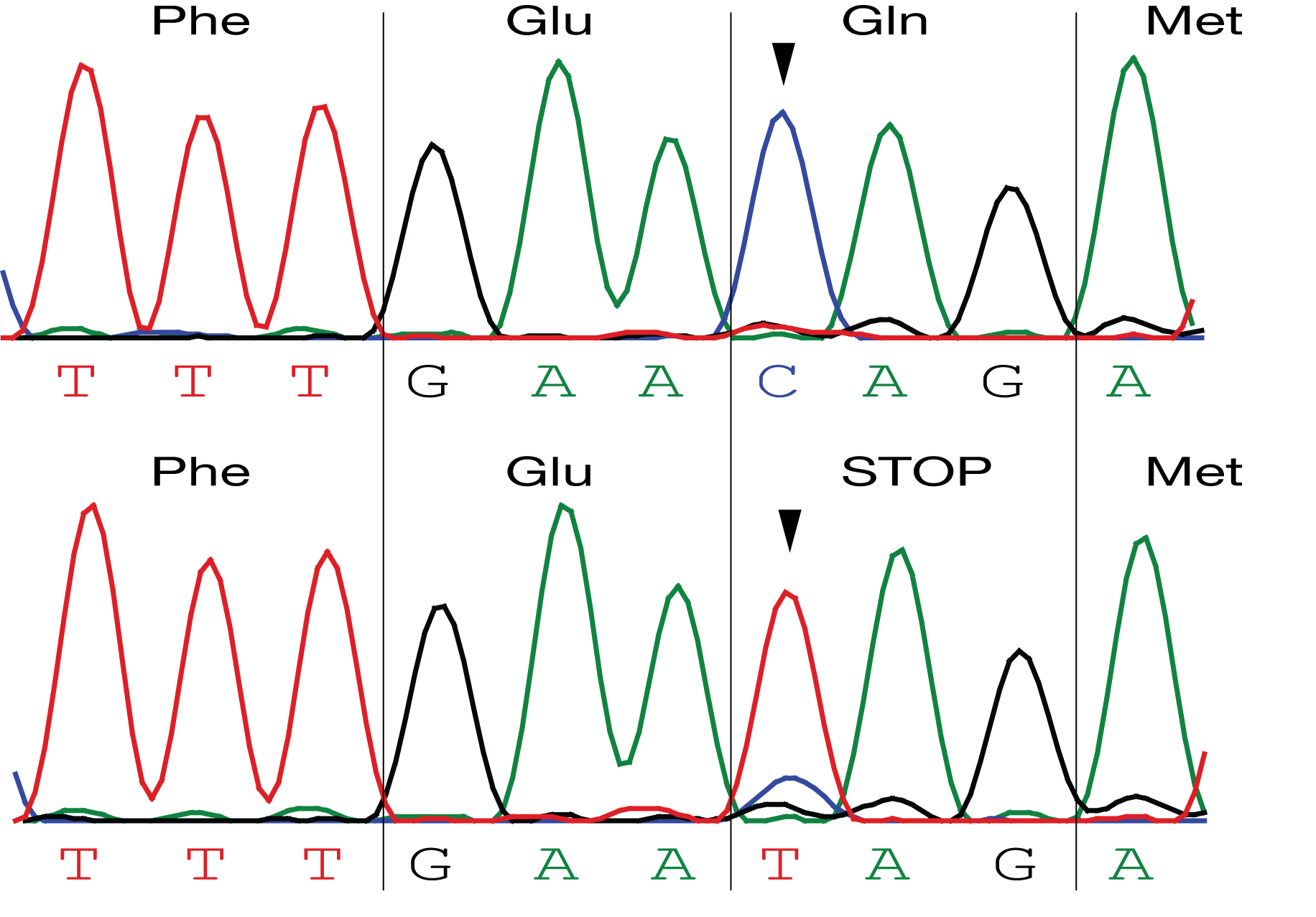

Supplement: Figure S2 — Sequencing of the cDNA of atp6v0d1 which lies near the cto locus in revealed a single nucleotide change that creates an early stop codon. (0.43 MB TIF) [file pgen.1000261.s002.tif]
