# Supplementary material for: Zebrafish Mutants calamity and catastrophe Define Critical Pathways of Gene–Nutrient Interactions in Developmental Copper Metabolism
Source: PLoS Genet. 2008 Nov 14;4(11):e1000261. doi: 10.1371/journal.pgen.1000261 (PMC2576455; doi:10.1371/journal.pgen.1000261)
Supplement: Text S1 — Supplemental methods. (0.03 MB DOC) [file pgen.1000261.s003.doc]

**Supplemental Methods:**

*Mutagenesis:* Male AB strain zebrafish at 12 weeks of age were placed in 3mM N-ethyl-N-nitrosourea (ENU) for 1 hour in the dark and then washed several times and allowed to recover for one week. This was repeated for a total of 5 exposures. The mutagenesis rate was monitored by crossing to *nacre* and scoring for mutant progeny. A rate of 1 *nacre* in 2000 embryos was obtained by this method. These mutagenized males were then crossed to AB females to generate the F1 generation carrying a random array of ENU induced mutations.

*Screen:* Clutches of eggs from F1 females heterozygous for random mutations were obtained and fertilized with UV-inactivated sperm to create gynogenetic haploid offspring (Fig 1A). At 3 hours post fertilization (hpf) half of each clutch was placed in 100nM neocuproine, a dose empirically determined to be a threshold dose for pigmentation defects in wild-type AB haploid embryos in our egg water (35mg/L Crystal Sea Marinemix, Marine Enterprises Int. Baltimore, MD with 18.3MΩ deionized water). At 48 hours post fertilization clutches treated with neocuproine were scored for loss of pigmentation. If the treated half of a clutch lacked pigment, the untreated half was examined. If this half also lacked pigment the mutant was discarded; if it contained any pigment at all, it was retained for characterization. F1 females from which mutant clutches were derived were out-crossed to AB for maintenance and crossed to WIK strain males for subsequent mapping. Mutants were confirmed by transmission of the phenotype to subsequent generations.

*Early Pressure Parthenogenesis and chromosomal assignment:* Gynogenetic diploid mapping has been previously described [1]. Briefly, clutches of eggs from heterozygous carriers were fertilized with UV-inactivated sperm and immediately placed in a french press. At 1’20” a pressure of 8000psi was applied to the eggs and held for 5 min. The embryos were then removed from the press and raised as normal larvae. Simple sequence length polymorphisms (SSLPs, <http://zebrafish.mgh.harvard.edu/>) near the centromere of each chromosome were used to assign the chromosome containing the mutation, as mutants will have no recombination between the mutation and the centromere. Also distance from the centromere is estimated based on the percentage mutants in clutches derived in this manner.

*Fine Mapping:* Haploid embryos were obtained as above from heterozygous AB/WIK females. At 48 hours post fertilization the embryos were sorted according to phenotype, the DNA was extracted individually using proteinase K/phenol/chloroform and known SSLP’s (<http://zebrafish.mgh.harvard.edu/>) were examined for linkage to the phenotype.

*Cloning:* Mutant *atp7a* was cloned and sequenced twice from a single cDNA preparation using *atp7a* specific primers. Wild-type and mutant *atp6v0d1* was similarly cloned using the following primers based on the database sequence (NM_199620).

*Allele-specific PCR:* Detection of the *cto^gw325^* mutation was accomplished using allele-specific PCR with the forward primer AAGCTTCCTGGCCAATGAAGC, the mutant specific reverse primer GATGTTAACAGCCTCCATCTA, and a positive control reverse primer CCAAATCCTGTTCAGAGATGC using a 62^o^ C annealing temperature with standard Taq polymerase (Promega).

1. Johnson SL, Africa D, Horne S, Postlethwait JH (1995) Half-tetrad analysis in zebrafish: mapping the ros mutation and the centromere of linkage group I. Genetics 139: 1727-1735.
